# Green microalga *Chromochloris zofingiensis* conserves substrate uptake pattern but changes their metabolic uses across trophic transition

**DOI:** 10.3389/fmicb.2024.1470054

**Published:** 2024-11-27

**Authors:** Yuntao Hu, Nakian Kim, Melissa S. Roth, Katherine B. Louie, Suzanne M. Kosina, Shivani Upadhyaya, Tim L. Jeffers, Jacob S. Jordan, Benjamin P. Bowen, Krishna K. Niyogi, Trent R. Northen

**Affiliations:** ^1^PrognomiQ Inc., San Mateo, CA, United States; ^2^Department of Plant and Microbial Biology, University of California, Berkeley, Berkeley, CA, United States; ^3^Environmental Genomics and Systems Biology, Lawrence Berkeley National Laboratory, Berkeley, CA, United States; ^4^Joint Genome Institute, Lawrence Berkeley National Laboratory, Berkeley, CA, United States; ^5^Department of Chemistry, University of California, Berkeley, Berkeley, CA, United States; ^6^Howard Hughes Medical Institute, University of California, Berkeley, Berkeley, CA, United States; ^7^Molecular Biophysics and Integrated Bioimaging Division, Lawrence Berkeley National Laboratory, Berkeley, CA, United States

**Keywords:** microalgae, *Chromochloris zofingiensis*, arginine, purine, metabolomics, orotic acid, trophic transition

## Abstract

The terrestrial green alga *Chromochloris zofingiensis* is an emerging model species with potential applications including production of triacylglycerol or astaxanthin. How *C. zofingiensis* interacts with the diverse substrates during trophic transitions is unknown. To characterize its substrate utilization and secretion dynamics, we cultivated the alga in a soil-based defined medium in transition between conditions with and without glucose supplementation. Then, we examined its exometabolite and endometabolite profiles. This analysis revealed that regardless of trophic modes, *C. zofingiensis* preferentially uptakes exogenous lysine, arginine, and purines, while secreting orotic acid. Here, we obtained metabolomic evidences that *C. zofingiensis* may use arginine for putrescine synthesis when in transition to heterotrophy, and for the TCA cycle during transition to photoautotrophy. We also report that glucose and fructose most effectively inhibited photosynthesis among thirteen different sugars. The utilized or secreted metabolites identified in this study provide important information to improve *C. zofingiensis* cultivation, and to expand its potential industrial and pharmaceutical applications.

## 1 Introduction

Photosynthesis and the central carbon metabolism of phytoplankton are crucial players in the global carbon cycle, as they are responsible for 50% of the global net primary production (Ryan-Keogh et al., 2023). Within this diverse polyphyletic group, microalgae occupy important niches in both aquatic and terrestrial ecosystems (Jassey et al., 2022). Microalgae have been widely studied because they are promising platforms to produce biofuel feedstocks and high-value natural products (Chia et al., 2022; Kumar et al., 2020). So far, various approaches have been taken to make microalgal products scalable and sustainable, including genetic engineering, innovative cultivation system design, and improved cultivation methods (Gupta et al., 2024; Jeffers et al., 2024; Rozenberg et al., 2024; Stavridou et al., 2024; Wang et al., 2024). Some oleaginous microalgal species can undergo a trophic transition between photoautotrophy and heterotrophy, depending on the availability of light and carbon sources. For example, *Auxenochlorella protothecoides* can quickly dismantle photosynthesizing organelles when given glucose in the dark and transition to heterotrophy, losing its green pigmentation (Shihira and Krauss, 1963). Using a different strategy, *Chlamydomonas reinhardtii* grows heterotrophically in the dark on acetate while maintaining photosynthetic organelles (Harris, 2009; Merchant et al., 2007). As demonstrated by these model species, trophic transition involves dynamic and strain-specific shifts in the microalgal morphology and physiology driven by complex metabolic changes. Exploring the complex mechanisms behind trophic transition may elucidate potential strategies to improve microalgal strains for biotechnological applications (Jeffers et al., 2024; Kong et al., 2020; Nicodemou et al., 2022; Roth et al., 2019b). Also, this feature can be employed to improve algal cultivation methods. For example, multi-step cultivation methods can couple carbon sequestration during photoautotrophic step with accelerated lipid production during heterotrophic step (Rismani-Yazdi et al., 2015; Zhang H. et al., 2021).

*Chromochloris zofingiensis* is an emerging model microalgal species that was first isolated from a soil in Switzerland (Dönz, 1934). This species is known to undergo trophic transitions in the light (Roth et al., 2019a), which entails dynamic adjustments to its metabolic processes depending on the available carbon sources (Zhang Z. et al., 2021). In a previous study, we observed that *C. zofingiensis* completely shuts down photosynthesis when given glucose under low iron conditions (Jeffers et al., 2024; Roth et al., 2019a, 2019b). This process included degradation of photosynthetic apparatus, changes in the thylakoid ultrastructure, and cell morphology, along with shifts in the gene expression associated with glycolysis, central carbon metabolism, and lipid biosynthesis (Roth et al., 2019a). Additionally, high amounts of iron rescue photosynthesis of *C. zofingiensis* leading to mixotrophic growth (Jeffers et al., 2024). Importantly, while growing on glucose, this species accumulates triacylglycerols (TAGs) and astaxanthin, both high-value coproducts (Breuer et al., 2012; Chen et al., 2023; Jeffers and Roth, 2021), supported by the transcriptomics results of their synthesis pathways. As a unicellular terrestrial alga, *C. zofingiensis* likely uses a diverse array of substrates in the soil environment, where glucose may be only one of many available heterotrophic substrates. Moreover, how this alga interacts with the various metabolites in the soil environment through uptake and secretion, and whether this differs by trophic modes, has not been investigated. Characterizing the exometabolic and endometabolic dynamics of this species in different trophic modes may provide useful information to improve its cultivation and expand its applications.

Here, we examined changes to the exometabolites and endometabolites of *C. zofingiensis* throughout the transition from the photoautotrophic (glucose-absent) condition to the heterotrophic (glucose-added) condition, and back to the photoautotrophic condition. We employed liquid chromatography tandem mass spectrometry (LC–MS/MS) based metabolomics analysis on both spent media and the cell pellets. To determine how this terrestrial algal species interacts with the common soil metabolites in trophic transition, we used a defined medium designed to included commonly detected soil metabolites, Soil Defined Medium (SDM), for culture growth (Jenkins et al., 2017). The substrate uses and secretion patterns of this species have not been determined before, especially in the context of trophic transition. Hence, our analysis using SDM may elucidate the substrates needs of this alga and how they change under different trophic modes. This information may further our understanding of *C. zofingiensis* to improve its cultivation method and expand its biotechnological applications. Then, we tested thirteen different sugars for their effectiveness to induce a transition to heterotrophy in *C. zofingiensis*. We found that arginine, lysine, and purines are preferred substrates of *C. zofingiensis*, and we report what is to our knowledge the first observation of orotic acid accumulation in the culture media by microalgae.

## 2 Materials and methods

### 2.1 Strain and growth conditions

*Chromochloris zofingiensis* strain SAG 211–14 was obtained from the Culture Collection of Algae at Göttingen University (SAG) (Friedl and Lorenz, 2012). The liquid cultures were cultivated in 125 mL Erlenmeyer flasks and maintained on a shaker (100–150 rpm) under diurnal illumination (100 μmol photons m^−2^ s^−1^ with 16:8 light and dark cycle) at 25°C. The cultures were grown in a modified SDM (Jenkins et al., 2017) supplemented with Wolfe’s vitamins and Wolfe’s mineral solutions (ATCC, Manassas, VA, USA). The SDM was chosen to test the substrate use and secretion patterns of *C. zofingiensis* for common soil metabolites. All sugars and sugar alcohols in the original recipe were excluded in the modified SDM. The total carbon content of the medium was 0.40 mM from the SDM metabolites and 1.87 mM from the vitamin supplements, too low to be a meaningful source of energy nor contribute to cell growth. The detailed composition of the modified SDM is described in Supplementary Table S1.

### 2.2 Trophic transition experiment

**Figure 1:**
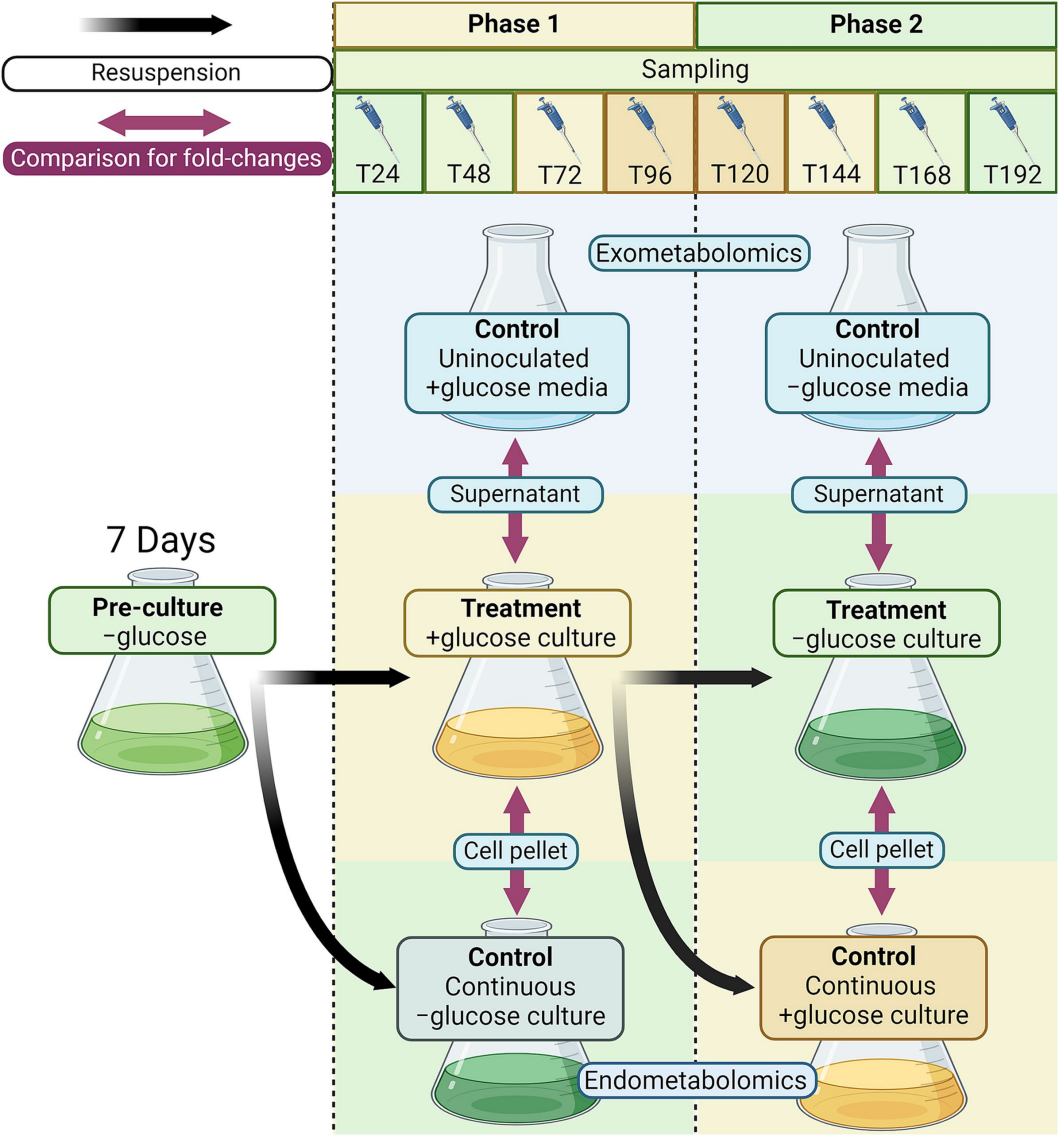
Experimental design for exometabolomics and endometabolomics of *C. zofingiensis* in trophic transition. Briefly, the −glucose pre-culture was resuspended into +glucose soil defined medium (SDM) and continuous −glucose SDM control. After 96 h, the +glucose culture was resuspended into −glucose medium for trophic transition treatment and into fresh +glucose medium for continuous heterotrophic control. Every 24 h since resuspension, and hour zero, samples were taken to measure cell density and diameter and perform metabolomics. All treatment and control cultures had three replicates. For exometabolomics, the fold-change difference between treatment cultures (to heterotrophy and back to photoautotrophy) and the uninoculated control media (SDM, incubated with the cultures) were calculated. For endometabolomics, the fold-change difference between treatment cultures (after −glucose pre-culture) and their respective control cultures (continuous −glucose, then continuous +glucose) were calculated.

A pre-culture of *C. zofingiensis* was grown in SDM as described above for 7 days to reach a cell density of 5 × 10^6^ cells mL^−1^, measured using the Multisizer 3 Coulter Counter (Beckman Coulter, Brea, CA, USA). For the first phase of the experiment (transition to heterotrophy; +glucose phase), the treatment cultures were prepared in triplicate by resuspending pre-culture cells into SDM supplemented with 40 mM glucose (+glucose culture; *n* = 3). Control cultures were prepared in triplicate by also resuspending pre-culture cells in SDM but without glucose (continuous −glucose control; *n* = 3). Samples were collected at 0, 24, 48, 72, and 96 h after the beginning of the experiment. The second phase of the experiment (transition back to photoautotrophy; −glucose phase) started after 96 h of the first phase. Each +glucose culture was aliquoted into two sets and cells were collected by centrifugation. Then, cells from one aliquot were resuspended into fresh modified SDM to induce transition back to photoautotrophy (−glucose culture; *n* = 3), and cells from another aliquot were resuspended into modified SDM with 40 mM glucose (continuous +glucose control; *n* = 3). Samples were collected at 120, 144, 168, and 192 h after the start of the experiment (or 24, 48, 72, and 96 h after the start of the second phase). Uninoculated, but incubated sterile medium controls were included in triplicate for all phases of the experiment. Throughout the experiment, all cultures were grown under the 16:8 light:dark cycle as described above. Samples were collected at the same time every day during the light cycle. The experimental design is visualized in Figure 1.

### 2.3 Photosynthesis suppression with thirteen sugars experiment

*Chromochloris zofingiensis* was grown in SDM as described above until the culture reached a cell density of 5 × 10^6^ cells mL^−1^. Then the culture was divided into forty-two 50 mL cultures (14 treatments x 3 replicates). For each treatment, each individual sugar was added to a final concentration of 240 mM carbon: 48 mM for each pentose (ribose and arabinose), 40 mM for each hexose (glucose, fructose, mannose, galactose, and rhamnose), 20 mM for each disaccharide (lactose, trehalose, maltose, cellobiose, and sucrose), and 13.3 mM for trisaccharide (raffinose). Maximum photosynthetic efficiency was measured as described above every 24 h after the sugar addition for 3 days.

### 2.4 Cell characterization

Maximum photosynthetic efficiency was measured with the FMS2 system (Hansatech Instruments, Pentney, UK). After cells were dark acclimated while shaking for 30 min, 3.5 × 10^6^ cells were collected onto a glass fiber filter which was then placed into the instrument’s leaf clip. The maximum efficiency of PSII, (*F*_m –_ *F*_0_)/F_m_ = *F*_v_/*F*_m_, was measured using a 0.5 s saturating pulse (at >2,000 μmol photons m^−2^ s^−1^). Cell number, cell mean diameter, and cell volume of the liquid culture were measured with a Multisizer 3 Coulter Counter (Beckman Coulter, Brea, CA, USA).

### 2.5 Metabolite extractions

Intracellular and extracellular metabolites were extracted for LCMS analysis. Cultures were spun down at 12,000 g for 5 min. The supernatant was transferred to a new tube and then both cell pellets and growth medium supernatants were lyophilized. Media controls were included for supernatants. Empty tubes were included as extraction blanks. The lyophilized cell pellets were bead milled, using 3.2 mm stainless steel beads three times, for 5 s at a time using a BioSpec Mini-beadbeater-96 (Bartlesville, OK, USA). Just prior to analysis, dried materials were resuspended in 150 μL of LCMS grade methanol containing internal standards (Supplementary Table S2). The samples were vortexed and centrifuged for 5 min at 5,000 g to remove insoluble material. Supernatants were filtered through 0.22 μm PVDF Millipore Ultrafree centrifugal filter tubes (5 min at 5,000 g). Filtrates were transferred to amber glass vials with caps for LCMS analysis.

### 2.6 LC–MS/MS

LC–MS/MS was performed on intracellular and extracellular extracts. Polar metabolites were separated with hydrophilic liquid interaction chromatography using and Agilent InfinityLab Poroshell 120 HILIC-Z column (2.1 × 150 mm. 2.7 μm) (Agilent, Hayward, CA, USA), with MS and MS/MS data collected using a Q Exactive Hybrid Quadrupole-Orbitrap Mass Spectrometer (ThermoFisher Scientific, Waltham, MA, USA). LC–MS/MS parameters are available in Supplementary Table S2. Samples consisted of four biological replicates each and four extraction controls, with sample injection order randomized and an injection blank run between each sample; internal and external standards were included for quality control purposes.

### 2.7 Data analysis and statistics

LC–MS/MS raw data were analyzed using MetAtlas (Yao et al., 2015). Briefly, samples were annotated by comparing mass-to-charge ratios (m/z), MS/MS spectra, and retention times (RT) with an in-house library of authentic reference standards. The metabolic pathways of the targeted metabolites were studied using the Kyoto Encyclopedia of Genes and Genomes (KEGG) (Kanehisa, 2019; Kanehisa et al., 2023; Kanehisa and Goto, 2000).

To calculate the fold changes of metabolites, the peak intensities of metabolites from treatment cultures (in trophic transition) were divided by those of their respective controls (replete media or continuous culture controls) and were log_10_ transformed. For all calculations, the peak intensities of the replicates were averaged for each treatment or control. For endometabolomics, the peak intensities of the cell pellets were normalized by their respective average culture volumes. All statistics and calculations were conducted with R (version 4.3.1) (R Core Team, 2023). The significance of fold changes between treatment samples and their respective controls were tested by non-parametric Kruskal–Wallis test, commonly used for metabolomics data that are often sporadic and skewed, at ɑ = 0.05. Figures were created in the R environment using the package *ggplot2* (Wickham, 2016).

## 3 Results and discussion

### 3.1 *Chromochloris zofingiensis* highly prefers exogenous lysine, arginine, and purines

**Figure 2:**
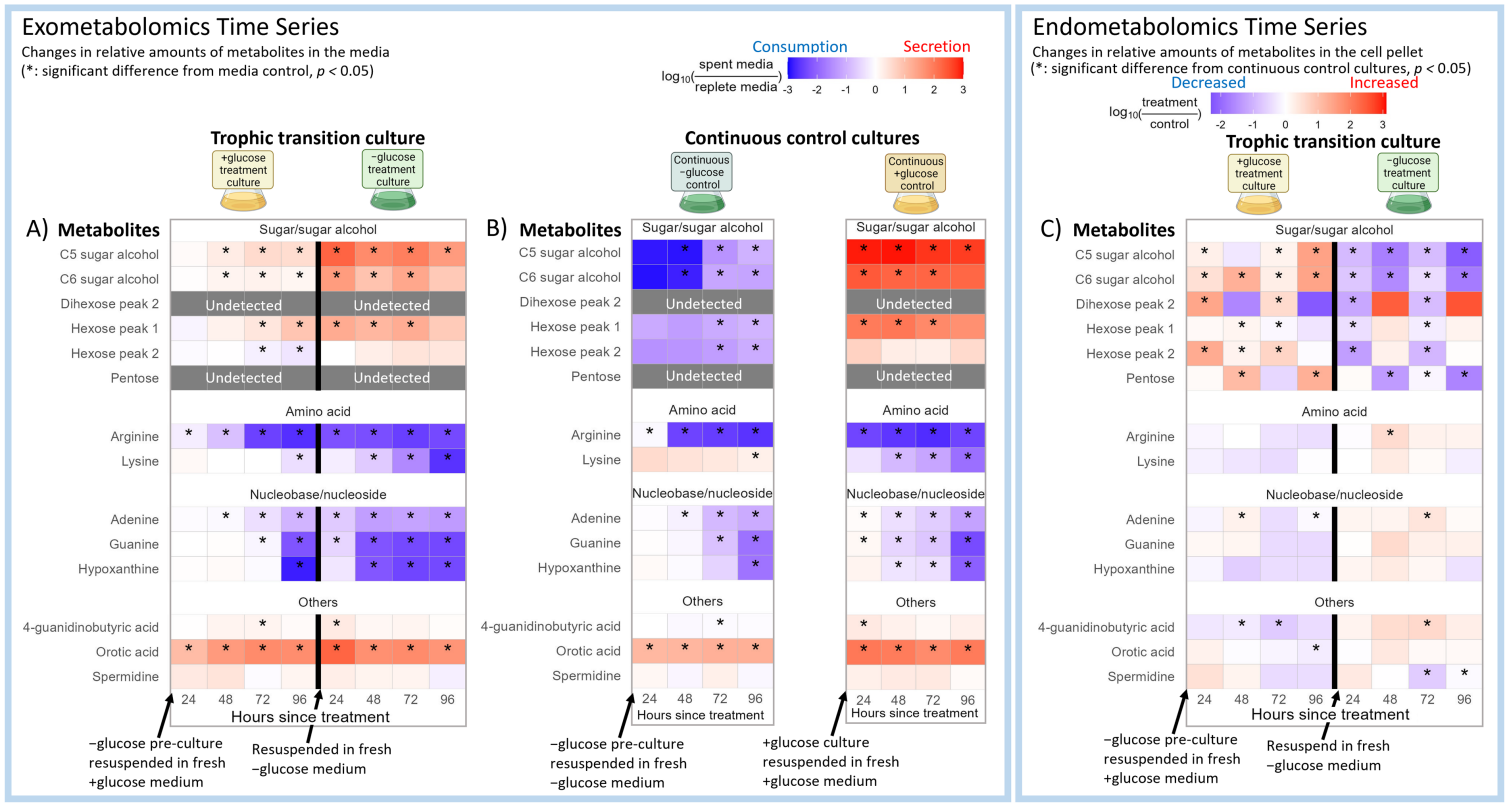
Time series of selected soil defined medium (SDM) metabolite profiles of *C. zofingiensis* for (A) exometabolites across transition from −glucose to +glucose condition (left), and back to −glucose (right), (B) continuous −glucose control (left) and continuous +glucose control (right), and (C) endometabolites across transition from −glucose to +glucose condition (left), and back to −glucose (right). The complete figure of all the SDM metabolites is in Fig. S1. The intensities of the endometabolites were normalized to the cell volume of each sample at the time of sampling. The log_10_ fold changes of the exometabolites between treatment cultures versus the replete defined media control, and those of endometabolites between +glucose phase versus continuous −glucose control, and − glucose phase versus continuous +glucose control are shown as a heatmap from positive (red) to negative (blue). The x-axis indicates the hours after the cultures were resuspended in fresh media to induce trophic transition. Tiles marked with asterisks had statistically significant fold change from the control (*p*-value <0.05). Gray boxes indicate metabolites that were undetected or had too much noise in the treatment cultures.

The trophic transition of microalgae involves morphological and physiological changes that may also involve significant shifts in substrate use or exudation patterns (Roth et al., 2019a; Zhang H. et al., 2021; Zhao et al., 2014). Since *C. zofingiensis* is a terrestrial alga isolated from soil, we examined its substrate use in a soil defined media (SDM) which is composed of many commonly detected soil metabolites. Overall, lysine, arginine, adenine, guanine, and hypoxanthine in the media showed statistically significant decreases (*p*-value <0.05) between about 10-fold and 700-fold during both +glucose and − glucose phases (Figure 2A). This pattern was also observed during the continuous −glucose and + glucose controls (Figure 2B). Lysine during continuous −glucose control was an exception as this pattern was not as prominent as it was for the other metabolites (Figure 2B). These results indicated that *C. zofingiensis* strongly prefers these five exogenous metabolites regardless of trophic modes, except for lysine during continuous −glucose control. Supplementary Figure S1 visualizes the fold changes of the complete list of SDM metabolites.

#### 3.1.1 Potential use of exogenous lysine for TCA cycle during transition to heterotrophy

**Figure 3:**
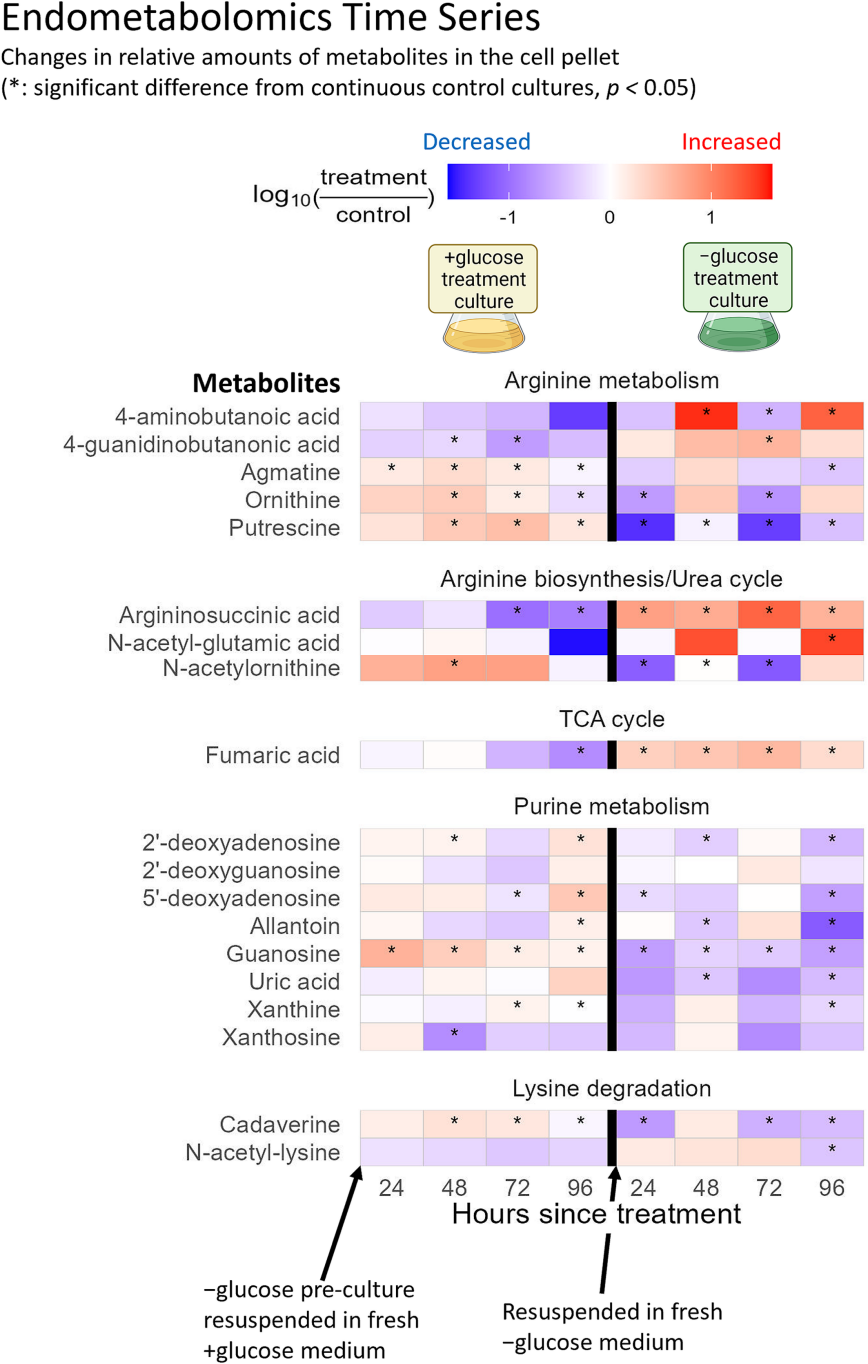
Timeseries of endometabolites, which were not part of the soil defined medium, of *C. zofingiensis* in transition from −glucose to +glucose condition (left), and back to −glucose (right). The intensities of the metabolites were normalized to the cell volume of each sample at the time of sampling. The log_10_ fold changes of the endometabolites between +glucose phase versus continuous −glucose control, and − glucose phase versus continuous +glucose control are shown as a heatmap from positive (red) to negative (blue). The x-axis indicates the hours after the cultures were resuspended in fresh media to induce trophic transition. Tiles marked with asterisks had statistically significant fold change from the control (*p*-value <0.05).

Among the twenty amino acids targeted in this study, *C. zofingiensis* showed strong preference for lysine and arginine uptake (Figures 2A,B). In contrast, the internal levels of lysine and arginine did not show consistent and significant increases (Figure 2C), suggesting that they are utilized upon uptake. Lysine and arginine uptake has been observed in diatom *Phaeodactylum tricornutum* (Flynn and Syrett, 1986; Yang et al., 2022), and improved algal growth with arginine supplementation has been reported (Acheampong et al., 2024; Bamary and Einali, 2023). During +glucose phase, we observed an intracellular increase in cadaverine, which is a product of the lysine degradation pathway that leads to the synthesis of succinate and acetyl-CoA and then enters the tricarboxylic acid (TCA) cycle (Figure 3). Meanwhile, N-acetyl-lysine, which is indicative of a different downstream pathway in lysine degradation, did not show consistent statistically significant changes (Figure 3). Indeed, lysine is a known energetic amino acid that provides electrons through the TCA cycle (Ingrisano et al., 2023). Thus, our results suggest that lysine might have been taken up by *C. zofingiensis* as a substrate for the TCA cycle to support its increased cellular activities due to the abundance of glucose.

#### 3.1.2 Differential role of exogenous arginine for putrescine synthesis during transition to heterotrophy versus TCA cycle during transition to photoautotrophy

Arginine may be a preferred substrate whose flows into different metabolic pathways differ between trophic conditions. Our metabolomics and past transcriptomics results suggest that exogenous arginine may support putrescine synthesis under +glucose condition, while it may support the TCA cycle under −glucose condition. The intracellular levels of ornithine, agmatine, and putrescine showed statistically significant (*p*-value <0.05) increase under +glucose condition compared to −glucose condition (Figure 3). In contrast, changes in the intracellular levels of the downstream products of putrescine metabolism, namely spermidine and 4-aminobutanoic acid, were not consistently significant (*p*-value >0.05) (Figures 2C, 3). Consistent with this finding, our previously reported transcriptomics data included upregulation of ornithine decarboxylase (Supplementary Figure S2), which catalyzes conversion of ornithine into putrescine, during the first 6 h of glucose supplementation (Roth et al., 2019a). These results support that the arginine taken up by *C. zofingiensis* during transition to heterotrophy enters the urea cycle or arginine metabolism to synthesize putrescine. Putrescine is known to be involved in plant development (González-Hernández et al., 2022). Also, exogenous putrescine has been shown to induce morphological and physiological changes in microalgae that are akin to those displayed during transition to heterotrophy (Freudenberg et al., 2022; Li et al., 2023; Roth et al., 2019a). The observed increase in intracellular putrescine under +glucose condition suggests that putrescine might play an important role in the microalgal transition to heterotrophy. Furthermore, we show that the alga likely synthesize the putrescine by metabolizing the taken up arginine.

Meanwhile, under −glucose condition, the intracellular levels of argininosuccinic acid and fumaric acid were significantly (p-value <0.05) higher than under +glucose condition (Figure 3). In the urea cycle, argininosuccinic acid can either metabolize into arginine or into fumaric acid, the latter of which enters the TCA cycle. Since the intracellular arginine level did not show clear signs of change under −glucose condition (Figure 2C), and since ornithine decarboxylase expression level was insignificant under the −glucose condition (Supplementary Figure S2), the exogenous arginine taken up by *C. zofingiensis* during transition to photoautotrophy may have completed the urea cycle to produce fumaric acid, which then entered the TCA cycle. Indeed, under −glucose condition, malate dehydrogenase and citrate synthase, which follows after fumaric acid in the TCA cycle, were somewhat upregulated, while pyruvate dehydrogenase was strongly downregulated (Supplementary Figure S2) (Roth et al., 2019a). These results suggest that *C. zofingiensis* may be using exogenous arginine as an important substrate to drive the TCA cycle when pyruvate from glucose is unavailable. The transitional use of exogenous arginine between +glucose and − glucose conditions may explain why arginine was consistently preferred by *C. zofingiensis* regardless of the trophic modes. Also, this demonstrates the dynamic metabolic acclimation of the cells to trophic conditions.

Overall, our metabolomics evidence, coupled with transcriptomics results from our previous studies (Roth et al., 2019a), showed that arginine and lysine had particular importance for *C. zofingiensis* to drive the TCA cycle under −glucose condition. Moreover, we also show that arginine might play a role in the algae’s cellular changes during transition to heterotrophy, via the putrescine synthesis pathway. Indeed, arginine supplementation has been shown to improve cultivation of other microalgal species (Acheampong et al., 2024). Thus, further investigation should test whether arginine and lysine supplementation can help improve the photoautotrophic cultivation of *C*. *zofingiensis*, which is a major challenge for the cost-effective applications of algae in biotechnology and sustainable energy. For example, if *C. zofingiensis* can procure arginine and lysine from cheap sources of amino acids such as animal or municipal wastes, the scalability could be improved for multi-purpose algal cultivation for natural products or biofuel production and wastewater treatment (Munz et al., 2020; Ummalyma et al., 2023). Similarly, further exploration on the role of putrescine in algal trophic transition might lead to new multi-step cultivation methods that seek to improve algal biofuel feedstock production (Ratnapuram et al., 2017; Zhang H. et al., 2021). For example, genetic modifications on putrescine synthesis might improve TAG production during photoautotrophy, if future studies confirm that putrescine is indeed involved in the physiological changes during heterotrophy, especially TAG accumulation.

#### 3.1.3 Increased use of exogenous purines for nucleotide synthesis and nitrogen acquisition

As for the strong preference for exogenous purines, their uptake and use by microalgae have been described previously (Shah and Syrett, 1984). Also, purines are known as important nitrogen reserves for many eukaryotic taxa like Chlorophyceae, which includes *C. zofingiensis* (Mojzeš et al., 2020; Pilátová et al., 2022). Our endometabolomics results indicated that the intracellular levels of a few metabolites within the purine metabolism and nitrogen recovery pathways – xanthine, uric acid, and allantoin—were generally inconsistent during the +glucose phase (Figure 3). However, they statistically significantly decreased (*p*-value <0.05) after transition to −glucose condition (Figure 3). Meanwhile, the intracellular levels of guanosine were significantly higher under +glucose condition, both during +glucose phase and continuous +glucose control, compared to their −glucose counterparts (Figure 3). These results suggest that *C. zofingiensis* is likely procuring N from purines and is accumulating more guanosine during transition to heterotrophy than to photoautotrophy. While guanosine synthesis might be indicative of increased nucleotide synthesis, further investigation is required. Also, we speculate that *C. zofingiensis* actively uses exogenous purines as nitrogen source during the transition to heterotrophy, especially when the culture has matured and rapidly exhausts other nitrogen sources. Along with the alga’s high preference for arginine and lysine, the observed preference for exogenous purines elucidated which substrates could potentially be used to improve *C. zofingiensis* cultivation. Further research should explore accessible sources of these substrates and how they can be incorporated into the cultivation methods.

### 3.2 Accumulation of orotic acid, sugars, and sugar alcohols in the media

We observed significant release of some metabolites into the media (Figures 2A,B). The extracellular level of orotic acid showed between 10- to 200-fold increases throughout the incubation for all treatment and control cultures (Figures 2A,B). The timeseries of orotic acid fold changes between individual cultures and the replete media control (Supplementary Figure S3) showed that its levels increased until the fourth day after the beginning of the experiment and plateaued with some fluctuations until the end of the incubation. This pattern was more consistent and stable with cultures in +glucose conditions, compared to −glucose conditions (Supplementary Figure S3). These results demonstrated that *C. zofingiensis* is likely secreting orotic acid up to a certain extracellular level and tends to maintain that level. Orotic acid is an intermediate of polyamine and pyrimidine synthesis and has been reported to be accumulated and secreted by fungi (Chiara et al., 2023; Nezu and Shimokawa, 2004; Silva et al., 2022; Swietalski et al., 2022). The endometabolite profile (Figure 2C) of *C. zofingiensis* did not show intracellular accumulation of orotic acid unlike these past reports. However, this species does have genes encoding dihydroorotase dehydrogenase, which catalyzes synthesis of dihydroorotate from glutamate metabolism into orotic acid (Roth et al., 2017). Nevertheless, this is the first report of orotic acid accumulation in the media by microalgae to our knowledge. Orotic acid has high pharmaceutical and industrial values as it is often used as a carrier in drug formulation and is an important substrate for pyrimidine production (Swietalski et al., 2022). Indeed, the reproducibility of our results should be tested, and further investigation is needed to understand why *C. zofingiensis* released orotic acid, and how this process is regulated. Yet, significant release of orotic acid into the media even under −glucose condition hints at potential biotechnological applications of this alga for orotic acid production for pharmaceutical applications and industrial pyrimidine production.

In the −glucose control culture spent media, sugars and sugar alcohols showed more than 800-fold decreases, suggesting strong uptake most likely to be spent as carbon sources or be stored (Figure 2B). Contrarily, strong exudation patterns were observed for these metabolites in the −glucose culture after transition from +glucose condition (Figure 2A). Sugar alcohols, both C5 and C6, showed a similar pattern as hexose (Figures 2A,B). Arabitol, one of C5 sugar alcohols, exudation has been reported in other organisms (Bowen and Northen, 2010), and both arabitol and mannitol, C6 sugar alcohol, are byproducts of glucose catabolism as observed in fungi (Adamczyk et al., 2023; Bernard et al., 1981; Diamantopoulou and Papanikolaou, 2023).

### 3.3 Endometabolomics of SDM metabolites of *C. zofingiensis* in trophic transition

Overall, the profile of SDM endometabolites in *C. zofingiensis* in trophic transition showed an opposite pattern between sugar and sugar alcohols versus other metabolites. The intracellular levels of sugars and sugar alcohols showed significant (*p*-value <0.05) increases during +glucose condition compared to continuous −glucose control; they decreased after transition to −glucose condition when compared to continuous +glucose control (Figure 2C). Meanwhile, other metabolites showed an opposite and weaker trend where their intracellular levels decreased during +glucose phase, while increasing after transition to −glucose phase. These results suggested that internal levels of sugars and sugar alcohols tend to be greater during the transition to heterotrophy, while other metabolites tend to be lesser. This result is consistent with the exudation patterns of the sugars and sugar alcohols during +glucose phase as seen in exometabolomics results (Figures 2A,B) and supports our speculation that they are secreted as overflow metabolites from the catabolism of glucose. The endometabolite trend was more consistent for serine, glutamic acid, and aspartic acid compared to other metabolites. Indeed, these are important amino acids that are associated with some steps of the TCA cycle (Chen and Wang, 2021). Furthermore, serine is an important source of one-carbon units used in various metabolisms like nucleotide synthesis (Reid et al., 2018). Our endometabolomics results suggest that the consumption of metabolites is increased in general during the transition to heterotrophy, leading to relatively lower intensities of non-sugar metabolites than those of photoautotrophic cells. This is potentially to support higher cellular activities due to the abundant carbon sources.

### 3.4 Repression of photosynthesis by different sugars

**Figure 4:**
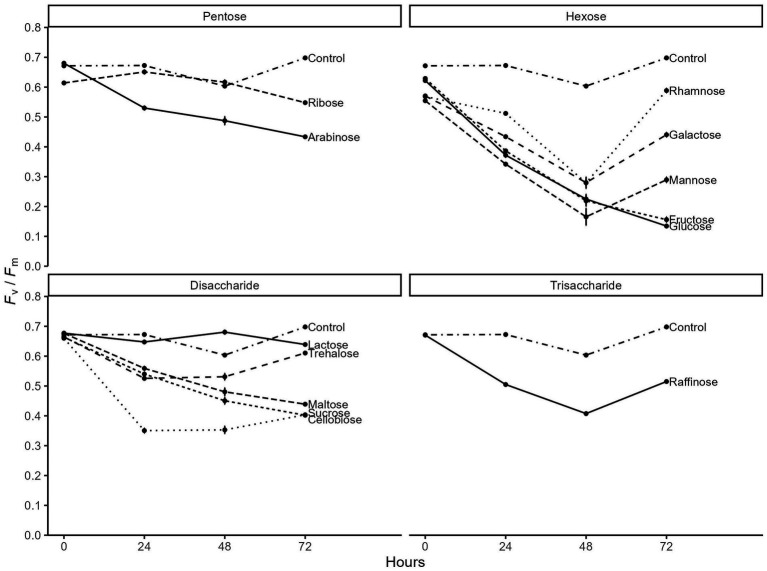
Time series of the maximum photosynthetic efficiency of photosystem II (*F_v_*/*F_m_*) of *C. zofingiensis* when given different sugars (*n* = 3). The x-axis indicates the hours since the start of experiment and the y-axis indicates *F_v_*/*F*_*m*._

Finally, we tested the effectiveness of different sugars to induce heterotrophy in the light for *C. zofingiensis* and confirmed glucose repressed photosynthesis the most. We compared the photosynthetic efficiency of the algae grown photoautotrophically versus grown on thirteen sugars individually. Compared to constant photoautotrophic control, all sugars except lactose decreased photosynthetic efficiency (*F*_v_/*F*_m_) to some level (Figure 4). Expectedly, glucose and fructose had the most consistent and dramatic effect on the *F*_v_/*F*_m_, reducing it from 0.62 down to 0.14 by day 3. While other hexoses, namely mannose, galactose, and rhamnose, showed comparable decrease in *F*_v_/*F*_m_ up to day 2, the maximum photosynthetic efficiency started to recover rapidly by day 3, suggesting that they may have exhausted the sugar. Pentose, disaccharides, and trisaccharides all showed less impact on *F*_v_/*F*_m_, with maximum reduction down to only around 0.4. While maltose, sucrose, and arabinose showed continuous decrease by day 3, *F*_v_/*F*_m_ either stabilized or recovered for all other pentoses, disaccharide, and trisaccharide. Overall, we found that glucose most dramatically and consistently induce the transition to heterotrophy in *C. zofingiensis*.

## 4 Conclusion

This study examined the uptake and exudation dynamics of metabolites by *C. zofingiensis* across trophic transitions. This alga displayed strong preferences for exogenous lysine, arginine, and purines. Orotic acid was secreted independently of trophic transition whereas sugar alcohol secretion depended on the glucose availability before the transition. Also, glucose was the most reliable elicitor of transition to heterotrophic growth among different sugars. Overall, this study characterized the dynamic exometabolomics and endometabolomics of *C. zofingiensis* across trophic transition. Further investigation employing transcriptomics and genomics is warranted to understand the metabolic pathways highlighted in this study, especially putrescine synthesis and the release of orotic acid. The highly preferred metabolites observed in this study – arginine, lysine, and purines – offer promising directions for future studies aimed at improving microalgal cultivation. Likewise, microalgal secretion of orotic acid has potential to further expand the pharmaceutical or biotechnological applications of *C. zofingiensis*.

## Data Availability

The original contributions presented in the study are included in the article/Supplementary material, further inquiries can be directed to the corresponding authors.
