# Correction: Green microalga *Chromochloris zofingiensis* conserves substrate uptake pattern but changes their metabolic uses across trophic transition

**DOI:** 10.3389/fmicb.2025.1641452

**Published:** 2025-07-02

**Authors:** Yuntao Hu, Nakian Kim, Melissa S. Roth, Katherine B. Louie, Suzanne M. Kosina, Shivani Upadhyaya, Tim L. Jeffers, Jacob S. Jordan, Benjamin P. Bowen, Krishna K. Niyogi, Trent R. Northen

**Affiliations:** ^1^PrognomiQ Inc., San Mateo, CA, United States; ^2^Department of Plant and Microbial Biology, University of California, Berkeley, Berkeley, CA, United States; ^3^Environmental Genomics and Systems Biology, Lawrence Berkeley National Laboratory, Berkeley, CA, United States; ^4^Joint Genome Institute, Lawrence Berkeley National Laboratory, Berkeley, CA, United States; ^5^Department of Chemistry, University of California, Berkeley, Berkeley, CA, United States; ^6^Howard Hughes Medical Institute, University of California, Berkeley, Berkeley, CA, United States; ^7^Molecular Biophysics and Integrated Bioimaging Division, Lawrence Berkeley National Laboratory, Berkeley, CA, United States

**Keywords:** microalgae, *Chromochloris zofingiensis*, arginine, purine, metabolomics, orotic acid, trophic transition

An incorrect Funding statement was provided. The Grant number DE-SC0023027 and the recipient institute The Regents of University of California, Berkeley, CA were erroneously omitted. The corrected funding statement appears below:

The author(s) declare that financial support was received for the research, authorship, and/or publication of this article. This material is based upon work supported by the U.S. Department of Energy, Office of Science, Office of Biological and Environmental Research, under Award Number DE-SC0018301, and DE-SC0023027 to The Regents of University of California, Berkeley, CA and DE-AC02-05CH11231 to Lawrence Berkeley National Laboratory including use of resources of the National Energy Research Scientific Computing Center, a Department of Energy Office of Science User Facility. KN is an investigator of the Howard Hughes Medical Institute. This article is subject to HHMI's Open Access to Publications policy. HHMI lab heads have previously granted a nonexclusive CC BY 4.0 license to the public and a sublicensable license to HHMI in their research articles. Pursuant to those licenses, the author-accepted manuscript of this article can be made freely available under a CC BY 4.0 license immediately upon publication. The graphical abstract was created with BioRender.com.

The original version of this article has been updated.

