# Supplementary figures and images for: Green microalga Chromochloris zofingiensis conserves substrate uptake pattern but changes their metabolic uses across trophic transition
Source: Front Microbiol. 2024 Nov 27;15:1470054. doi: 10.3389/fmicb.2024.1470054 (PMC11631937; doi:10.3389/fmicb.2024.1470054)

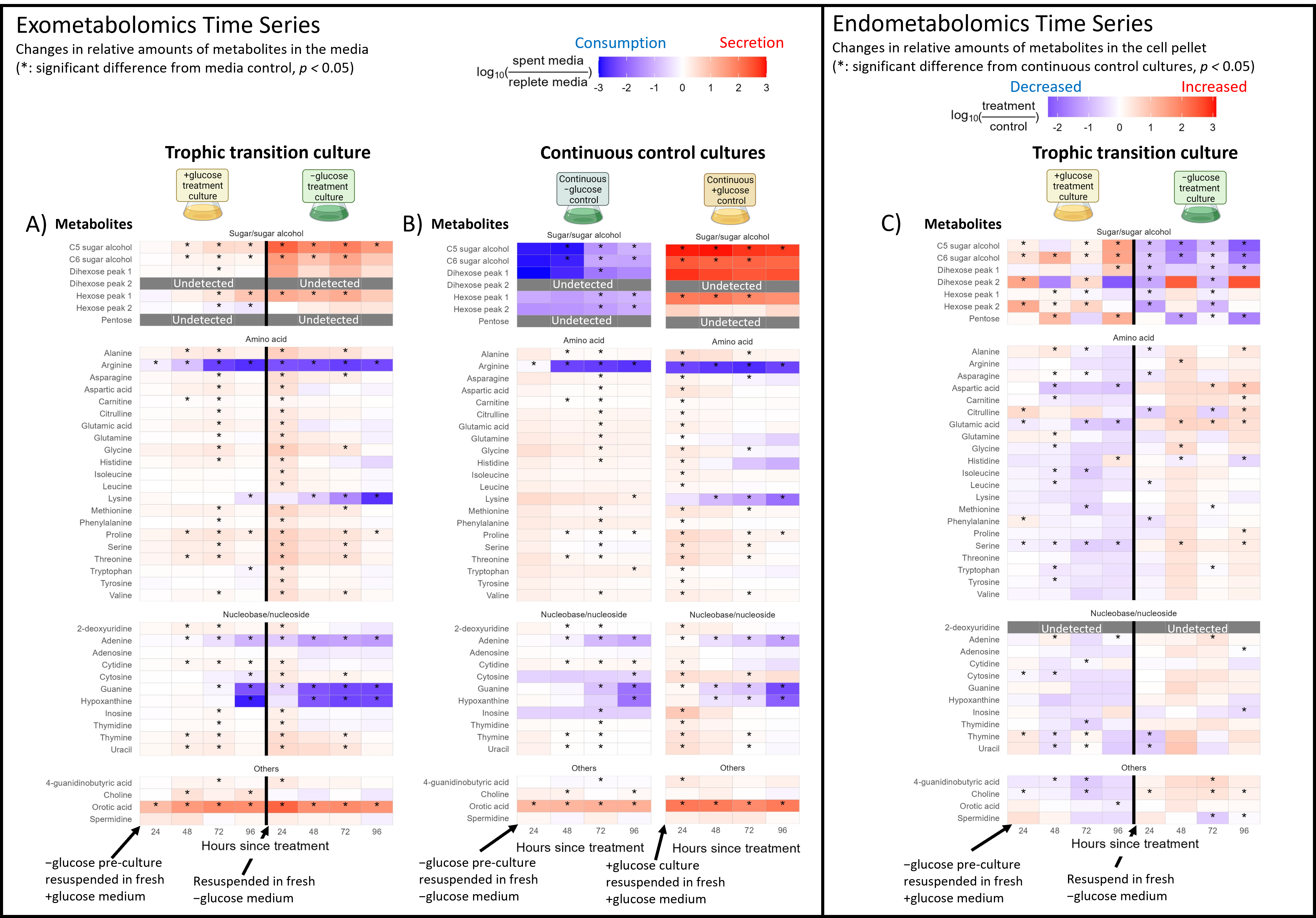

Supplement: Supplementary file 1 [file Image_1.tif]

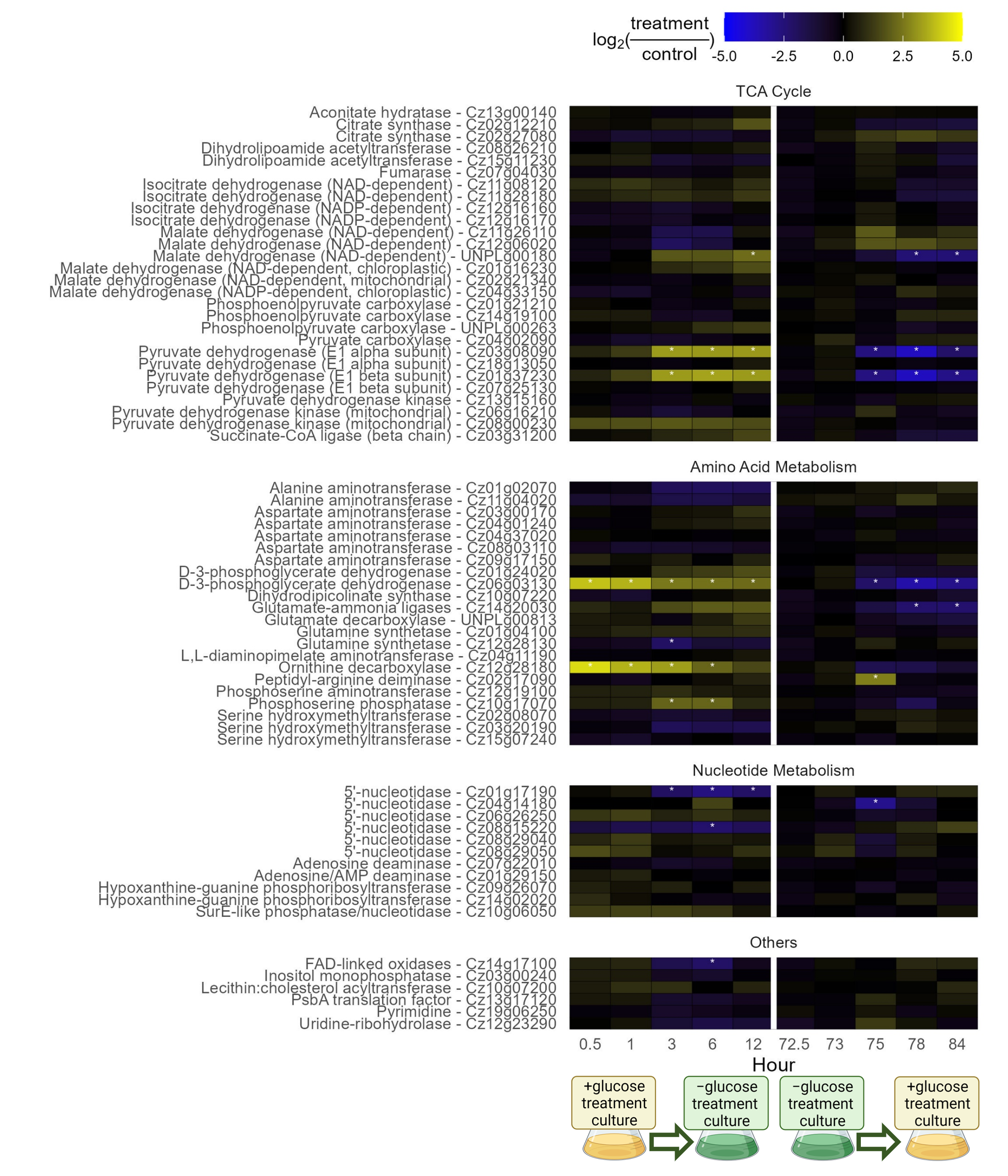

Supplement: Supplementary file 2 [file Image_2.tif]

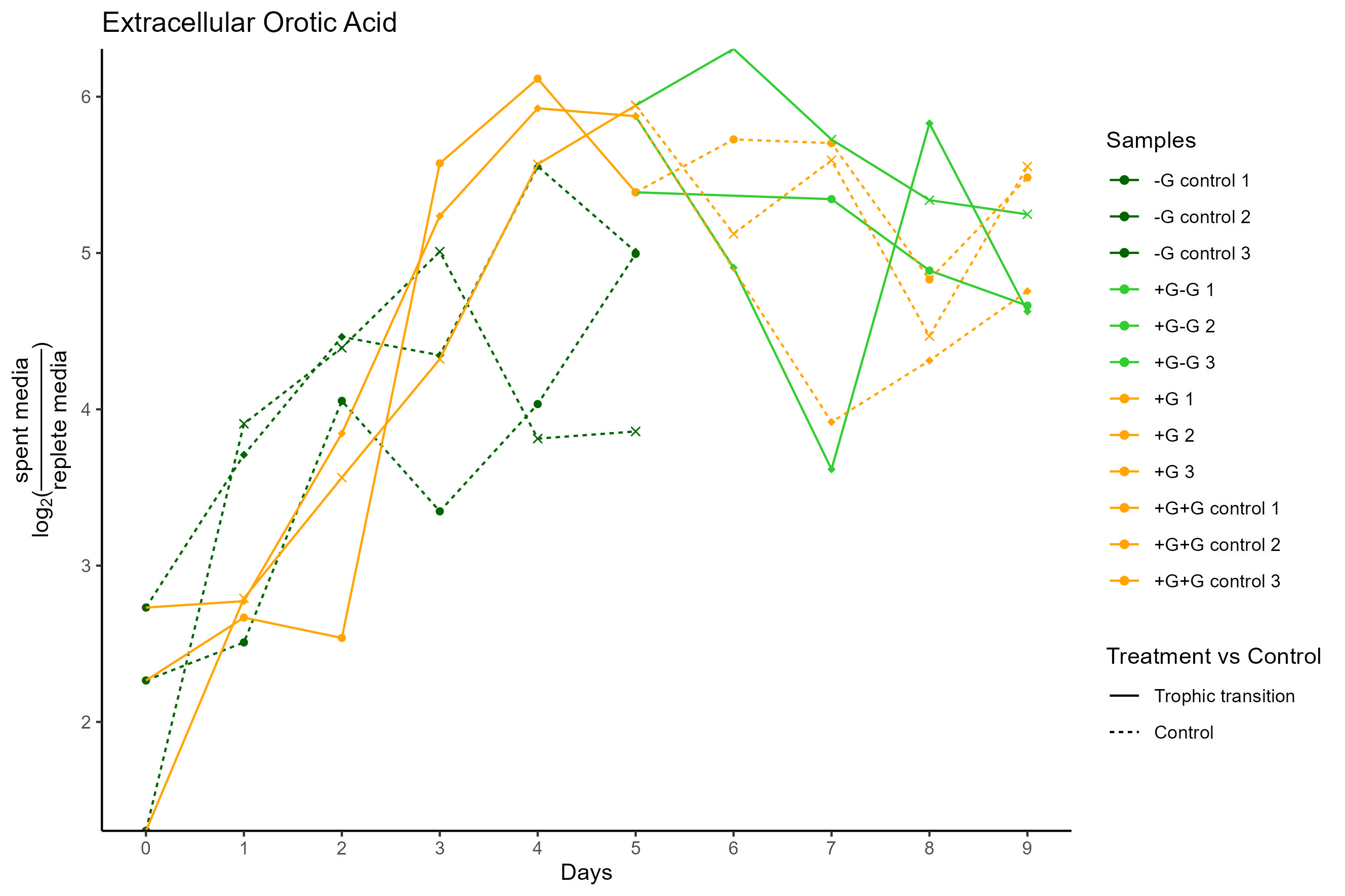

Supplement: Supplementary file 3 [file Image_3.jpeg]
